# Salutatory

**Published:** 1876-10

**Authors:** 


SALUTATORY.—After several years absence from the editorial chair, circumstances have transpired making it necessary for me to assume the direction of this journal again. This is done with a full knowledge of the laborious responsibility attached, and not without misgivings as to the power of catering to the varied tastes of readers, and at the same time sustain an elevated standard of medical literature. We shall spare no pains however, to please those who desire the advancement of scientific practical medicine.

The JOURNAL is intended for, and offered as a medium of communication between members of the medical profession, and to this end original articles are solicited from practitioners throughout the country. While theoretical reasoning should not be discarded, we must insist on the importance of practical facts as the best means of promoting professional advancement in the cause of humanity. Many valuable facts discovered by members of the profession who are disinclined to write articles for publication, lie forever hidden with the discoverer. Short communications, requiring very little sacrifice of time or labor, add greatly to our knowledge of practical medicine. Such are desirable for medical journals, and it is a duty we owe the profession and the public to furnish them for publication.

The JOURNAL, as heretofore, will recognize a high rational standard of medical ethics, and retain its life-long motto of "peace and science, but truth without fear."

While its pages will be open to the discussion of questions connected in any way with the honor and dignity of the profession, yet individual members or combinations will not be allowed this as a medium to attack their brethren through personal pique.

Arrangements have been made by which the proprietorship of the JOURNAL has passed into the hands of the publishers, Messrs. Dunlop & Dickson, with whom all settlements of dues and outstanding debts must be made. Their successful effort hitherto in producing a publication every way respectable in appearance, is a sufficient guaranty of faithfulness in the future.

J. G. WESTMORELAND, M.D.

In assuming the associate editorship of the JOURNAL, I feel no littte diffidence, from the limited experience I possess in journalism, and offer only the assurance of an earnest and persevering effort to discharge my duty. In the task of proof-reading and making selections, it will require but little more of ingenuity than I may naturally have. For any duty requiring originality, which it becomes my part to assume, I must crave the indulgence of the readers of the JOURNAL. To make the JOURNAL a first-class medium of information shall be the earnest effort of the editors, and to this end they should have the full co-operation of the profession. This can be fully consumated only by each one communicating any matter that, in his judgment, would be of interest.

Just at the present period of centennial jubilee, many very interesting discussions are taking place in the various medical associations. Reports of these we shall attempt to give from time to time, and the readers of the JOURNAL will find many points of interest to be found therein.

In addition, we solicit reports of the various local societies, and hope thereby to constantly furnish information as to the status of the profession at large.

In conclusion, we earnestly ask the aid of all those feeling an interest in the profession to contribute to the dissemination of information throughout the land.

R. W. WESTMORELAND, M.D.

